# Artificial Neural Networks-Based Material Parameter Identification for Numerical Simulations of Additively Manufactured Parts by Material Extrusion

**DOI:** 10.3390/polym12122949

**Published:** 2020-12-10

**Authors:** Paul Meißner, Hagen Watschke, Jens Winter, Thomas Vietor

**Affiliations:** Institute for Engineering Design, Technische Universität Braunschweig, Hermann-Blenk-Strasse 42, 38108 Brunswick, Germany; h.watschke@tu-braunschweig.de (H.W.); jens.winter@tu-braunschweig.de (J.W.); t.vietor@tu-braunschweig.de (T.V.)

**Keywords:** parameter identification, direct inverse model calibration, machine learning, hyperparameter optimization, feedforward artificial neural network, modeling strategy, additive manufacturing

## Abstract

To be able to use finite element (FE) simulations in structural component development, experimental investigations for the characterization of the material properties are required to subsequently calibrate suitable material cards. In contrast to the commonly used computational and time-consuming method of parameter identification (PI) by using analytical and numerical optimizations with internal or commercial software, a more time-efficient method based on machine learning (ML) is presented. This method is applied to simulate the material behavior of additively manufactured specimens made of acrylonitrile butadiene styrene (ABS) under uniaxial stress in a structural simulation. By using feedforward artificial neural networks (FFANN) for the ML-based direct inverse PI process, various investigations were carried out on the influence of sampling strategies, data quantity and data preparation on the prediction accuracy of the NN. Furthermore, the results of hyperparameter (HP) search methods are presented and discussed and their influence on the prediction quality of the FFANN are critically evaluated. The investigations show that the NN-based method is applicable to the present use case and results in material parameters that lead to a lower error between experimental and calculated force-displacement curves than the commonly used optimization-based method.

## 1. Introduction

In recent years, the field of additive manufacturing (AM) or 3D printing has become increasingly important. AM enables products with complex shapes and a high level of functional integration. This has led to new freedoms in design, which in turn require new design principles [1,2]. An advantage of additive manufacturing is a significant reduction of time-to-market, since no complex and cost-intensive tools must be developed and manufactured for the production and subsequent testing of a part. In contrast, high process- and machine-specific influences on the mechanical properties of the material represent a challenge for AM [3]. However, in order to design a prototype in the early stages of development, which is then tested under realistic conditions, suitable materials must first be selected, and a part dimensioning must be accomplished. Due to the complexity caused by structural load cases on industrial parts, FE simulations are usually used for this, which require respective material cards to describe the material-mechanical properties. The calibration of the material models (material parameter identification) is typically performed by a trial-and-error approach or by a semi-automatic iterative optimization process and requires a high degree of expert knowledge [4,5,6]. Furthermore, this commonly used process is computational- and time-expensive and thus cost-intensive. Especially in early phases of the product development process, different construction materials are often compared or subsequently modified, which requires the use of recalibrated material cards for the numerical simulation. In the commonly used PI process, the complete iterative calibration process must be repeated, even if only minor changes are made to the material to be calibrated—e.g., due to a changed amount of a material additive. This circumstance strongly limits the reduction of the product development time.

As an alternative and novel method for PI, a machine learning neural network (NN)-based approach can be used in a direct inverse process to determine the material parameters (physically or just mathematically-based parameters of the material model) that best fit the experimental data [7]. A major advantage of this method lies in the reusability of the NN previously trained for a specific material model to identify parameters for altered materials with similar material properties. This enables both the efficient calibration of material cards for similar materials as well as for different machines and process settings. In this way, the development time would be reduced and the accuracy in the design of parts could be increased. The repeated prediction for different materials certainly requires the ability of the material model applied to reproduce the material characteristics with sufficient accuracy. Furthermore, it is also necessary that the input, employed for training, sufficiently covers the range of characteristics of the material used. The material parameters can then be determined immediately in a time-saving process and an objective comparison of different materials in early phases of product development is enabled.

In the literature, different ML techniques are presented [8,9]. In this paper, methods for creating such an artificial neural network (ANN) (see Section 2.2) and the subsequent PI process for the application of an additively manufactured ABS under uniaxial tensile load are explained, compared and critically discussed. The used methods belong to the group of supervised learning. In this field of ML an algorithm is trained on a group of data, in which each data set contains a label. This label signifies a particular class or value that the training point belongs to [8]. The input of the training data set is fed into the input layer of the network and subsequently the states of the output neurons are compared to those states of the target values. The weights and the thresholds (biases) are changed to minimize the differences between network outputs and targets for all input patterns in the training set [9]. In this way, the network learns to predict input features for desired outputs of unseen data.

Contrary to the other methods exemplarily presented in [8], in which ML algorithms were used to completely replace FE simulations and to directly predict the stress-strain behavior, a different approach is used in this paper. In this case, a ML-based approach with neural networks is applied to determine the required material parameters of the chosen material model for the FE simulation as efficiently and precisely as possible. In the most common material PI procedure, a certain error measure between the numerical and experimental data set is defined, which for example could be load-displacement curves, stress-strain curves, eigenfrequencies or eigenvalues. The aim of the parameter identification procedure is then to minimize this error by modifying of the material parameters [10,11] in an analytical calculation and/or numerical simulation using different optimization procedures such as gradient-based methods [4,5,12,13,14] or genetic algorithms [15,16]. However, the success of optimization algorithms is often depending on the chosen starting points, which are usually unknown in advance. In order to find appropriate parameters for complex material models, typically many iterations are required, which increases calculation costs and often results in finding only local optima. Furthermore, the complete PI procedure must be started from scratch, if material parameters for (even slightly) different materials must be identified. Through this identification procedure, a set of parameters for the numerical model is finally obtained, which is suitable in combination with the appropriate material model for representing the material behavior in the numerical simulation. For this standard identification procedure usually user-friendly software tools (e.g., *LS-OPT*, *Valimat*) are used. In [17] a general overview of different methods for PI is given.

In contrast, the approach to train a FFANN using a squared loss function to predict the material parameters for the material model directly was first described by Yagawa et al. [18]. In this approach the training data are generated by the FE simulation respectively the material model itself and consists of material model parameters and their corresponding responses. Since in this approach a NN (typically a FFANN) is trained to the reverse input/output relations, this is also called a direct inverse model [19]. Yagawa et al. used this approach to identify parameters of a visco-plastic material model and since then this approach has been applied to different materials, respectively material models. Huber and Tsakmakis identified the material parameters for pure kinematic hardening inplasticity laws [20] and plasticity with non-linear isotropic and kinematic hardening [21] with this approach. Lefik and Schrefler estimated the parameters of an elasto-plastic model using an ANN for the FEA of a superconducting cable [22]. Nardin et al. used the approach for the identification of parameters of a constitutive law for soils and trained the ANN using the results from macro- and micro-mechanical numerical models [23]. Using a NN, Helm determined the parameters of a self-developed phenomenological system of constitutive equations to describe the pseudoelastic material behavior of NiTi shape memory alloys [24]. Chamekh et al. showed that using suitable ANN with this direct inverse approach, material parameters for describing anisotropies can also be identified by determining the anisotropic HILL law parameters [25]. Aguir et al. presented the advantage of this NN-based method in the low CPU time compared to the standard optimization-based method [26]. Therefore, they identified the parameters for the Hill’48 criterion under the associative normality assumption and the Voce law hardening parameters of the stainless steel AISI 304 as well as the orthotropic criterion of Hill’48 under the non-associative normality assumption. In [11] improvements for this direct inverse approach have been proposed by Unger and Könke using a Bayesian neural network [27] to additionally verify the accuracy of the identified parameters and their correlation. Morand and Helm investigated in [6] problems occurring if the PI problem is non-unique and developed an approach using a mixture of expert model to partition the non-unique problem in subtasks. In [28] different strategies for calibrating non-linear mechanical models with NN are reviewed. Also, their advantages and disadvantages are demonstrated using a calibration of four parameters for an affinity hydration model.

The current work addresses the application of neural network-based PI approaches to an additively manufactured test specimen using material extrusion made of ABS and the validation of its suitability. For this purpose, tensile test specimens (DIN EN ISO 527-1) were additively manufactured and afterwards mechanically tested. In this initial work, the elastic-plastic material model *MAT024* [29] was used in *LS-DYNA* and mechanisms such as damage and strain rate dependence were initially neglected. Therefore, it was possible to keep the number of material parameters to be identified small and yet to reproduce the complex material behavior of the thermoplastic ABS under uniaxial static tensile load in a structural simulation. Since the investigated ABS exhibits a pronounced softening, a self-developed custom formulation was used instead of yield curves known from literature and mainly used for metals, like Hockett–Sherby. This formulation consists of a combination of a second-degree polynomial and a root function and contains four parameters to be calibrated a,b,c,d:

(1) $$\sigma =a*\varepsilon_{pl}^{2}+b*\varepsilon_{pl}+c+d*\sqrt{\varepsilon_{pl}}.$$

These four material parameters were determined using both the commonly used iterative optimization procedure with the software *LS-OPT* and the NN-based parameter identification approach. Both approaches were then compared with each other in Section 2, with the latter being explained in more detail. Furthermore, methods for data generation and modification as well as different modeling techniques of NN’s are presented, which aim to achieve a higher precision of the predicted material parameters. The focus of the paper is to quantify and qualitatively evaluate the relationships in the modeling strategies for NN’s regarding the use case of structural simulation of additive processed thermoplastics. The aim is to build up NN’s with minimum effort and maximum prediction quality for PI. The research results are presented and critically evaluated in Section 3. In addition, different sampling strategies for the generation of necessary training data will be discussed. Since the hyperparameters of the NN have a significant influence on the prediction accuracy, different strategies such as gridsearch, randomsearch and HP optimization—using different optimization algorithms—were evaluated and compared. In Section 4 the results are finally summarized and an outlook on further research gaps is given.

## 2. Materials and Methods

In this section, the two methods for material parameter identification are presented first, whereas the NN-based method is discussed in more detail. Subsequently, the structure of the FFANN and the methods used are briefly presented for a better understanding of the latter approach. Furthermore, the data generation procedure for the training of the NN’s as well as the experimental characterization of the additively manufactured test specimens are presented.

### 2.1. Material Parameter Identification

Through the PI process, the required material parameters for the, in most cases, highly non-linear material models are determined, and thus calibrated material cards are generated. The aim of the model calibration is to estimate unknown parameters knowing the experimentally obtained response of a system to the given load conditions. However, this presupposes that the material model used is basically capable of reproducing the material behavior with sufficient accuracy through its mathematical formulation. Together with the numerical model, able to correctly simulate the experiment (EXP), the effective and robust PI method is essential for structural modeling and reliability assessment. To solve this identification problems there are two main methods, as mentioned before in Section 1. The most broadly used approach to PI is usually done by an error minimization technique, where the distance between the parameterized model predictions and the experimental test results is minimized [17]. However, this error minimization technique often leads to difficult optimization problems, which are highly non-linear and multi-modal. The second approach, a direct inverse NN-based procedure, assumes the existence of an inverse relation between outputs and inputs. The retrieval of desired inputs takes only a few seconds and could be easily executed repeatedly, if such a relationship is established. Within the several last years, a lot of attention was paid to the so-called intelligent methods of information processing and among them especially to soft computing methods, such as ANNs. In the following, both approaches are presented in more detail.

#### 2.1.1. Iterative Optimization Procedure

An iterative optimization procedure for PI is defined as the minimization of an error function F(x) specified as the difference between the outputs of the model $y^{M}$ and the output of the experiment $y^{E}$ (measurements in form of stress-strain or force-displacement-curves), i.e.,

(2) $$minF\left(x\right)=min||y^{E}-M\left(x\right)\left|\right|;$$

(3) $$y^{M}=M\left(x^{M}\right).$$

In this context, *M* represents the material model with its constitutive relations, which describes the stress-strain relationships and *x* the unknown parameters for the material model. A solution $x^{M}$ comes with the minimum of this function and if $F(x^{M})>0$, the remaining error is caused by inaccuracy of a model or by some noise in the measured data [10].

Like mentioned before, Equation (2) is classically solved by gradient-based optimization methods or genetic algorithms. Disadvantages of this method are:

- The success of optimization algorithms is highly depending on the chosen starting point, which is usually not known
- Many iterations are needed to find appropriate parameters for complex material models, which leads to high computational costs
- A high number of error function evaluations are needed
- Even for small changes in the experimental setup or the examined material the computationally and time-consuming search must be repeated [6,10]

As mentioned in Section 1, the software *LS-OPT* was applied to the presented problem in this paper as an iterative optimization procedure for comparison purposes as well as proof of the applicability of the custom approach for the description of the yield curve. In order to get the best possible setting of the searched parameters with *LS-OPT*, the sequential response surface method was used. For further information regarding PI problems with *LS-OPT*, see [30].

**Figure 1:**
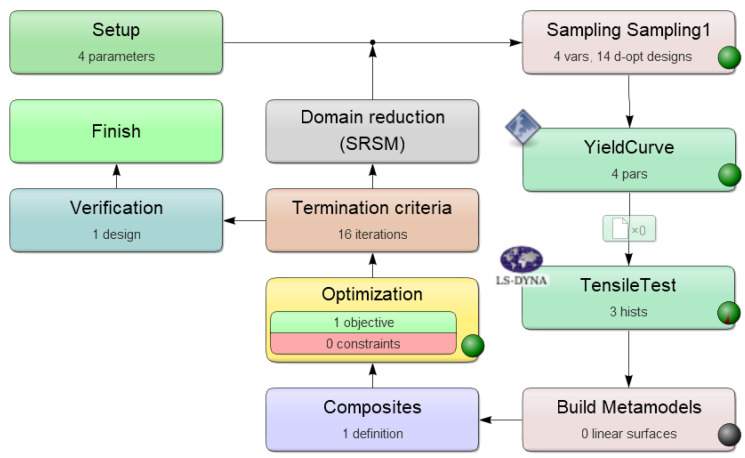
Parameter identification process to obtain the four material parameters for the custom yield function of *MAT024* with LS-OPT.

Figure 1 shows the optimization process to identify the four material parameters (a,b,c,d) with *LS-OPT* as it is displayed in the GUI of this optimization tool.

The goal of this procedure is to optimize the four material parameters to fit experimental tensile test data i.e., to minimize the difference between the experimental and the simulation force-displacement-curves (FDC) in a least square sense. However, the four material parameters are not an explicit part of the material model (*MAT024*) in *LS-DYNA*, instead the yield curve and the corresponding parameters are used to generate value pairs (stresses vs. plastic strain) in tabular form, which are then imported into the *MAT024*-card. In order to integrate this into the *LS-OPT* optimization process, a Python script was created which automatically generates the yield curve as a function of the four material parameters and then exports it as an ASCII file. Inside *LS-OPT* these four parameters were then stored as variables, which must be optimized. Furthermore, a FE-model of the tensile test was created (see Section 2.3) and imported into *LS-OPT*. To ensure the comparability with the direct NN-based procedure, the same FE-model with the same discretization was used. Furthermore, a maximum of 16 iterations with 14 simulation points per iteration and the default value 0.01 for the design change tolerance as well as the objective function tolerance were used. Mean squared error (MSE) was used as a method to compare the FDC. The results are presented in Section 3.1.

#### 2.1.2. Direct Neural Network-Based Procedure

**Figure 2:**
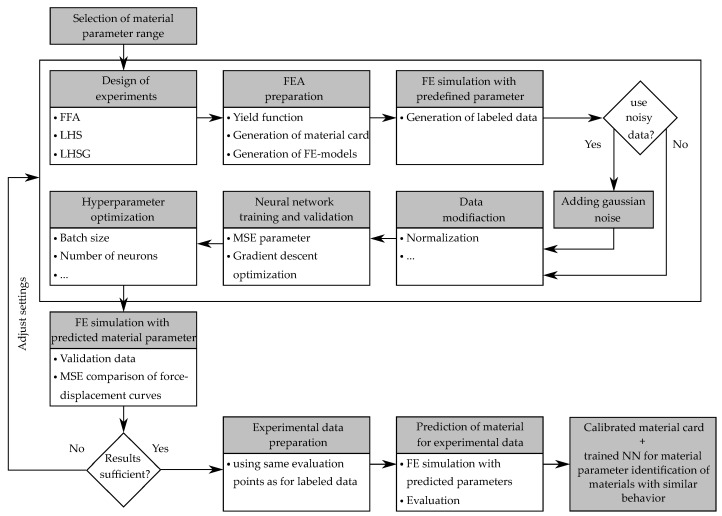
Workflow of the used direct NN-based parameter identification procedure applied to the problem of identifying the material parameters of the custom yield curve formulation for *MAT024*.

The direct inverse neural network-based procedure, assumes there is an inverse model $M^{INV}$ associated with the model *M*, which fulfils the following equation:

(4) $$x=M^{INV}\left(y\right)$$

for all possible *y* [10]. The main disadvantage of this approach is the extensive search for the inverse relationship. In contrast, the main advantage lies in the retrieval of the desired inputs in just a few seconds. During the training process, all the information generated so far (input—output relationship) are fed into the NN. Contrary to the iterative optimization-based method with gradients or genetic algorithms, decisions are always made based on current designs. The application of this direct inverse approach for the identification of material parameters of a visco-plastic material model was first described in [18]. The core of this method is the training of a NN to directly estimate the material parameters for a certain input (e.g., force-displacement curves). Figure 2 shows the workflow of this method applied to the explained problem of identifying the material parameters of the custom yield curve formulation for *MAT024*.

The shown workflow was implemented in a self-developed Python (V.3.7) environment. The libraries *Tensorflow* (V.2.1.0) and *Keras* (V.2.3.1) were used to create the NN’s and implemented into the workflow. The calculations based on NN’s were executed on a GPU.

In this PI procedure the necessary training data are generated by the material model itself and consist of material model parameters and their corresponding responses. In a first step the material model is analyzed, and suitable parameter ranges are defined to cover the material behavior. With suitable sampling methods the material parameter combinations are generated. To achieve the maximum possible prediction quality of the trained NN, it is necessary that the training data used are representative. The input data must cover the input space sufficiently and should not contain too many examples of one type at the expense of another. A variety of different sampling methods with advantages and disadvantages exist. As an example, the *Full Factorial Approach* (FFA), *Monte Carlo Methods*, *Hammersly Methods* are mentioned, which will not be discussed in detail. In this paper three different DOE methods were used: FFA, *Latin Hypercube Sampling* (LHS) [31] and *LHS with genetic space filling* (LHSG) [32]. Furthermore, their influence on the resulting prediction quality of the FFANN for the present problem were investigated (see Section 3.2.1). The Python library *PyDOE* was used for the LHS and the library *Nodeworks*, developed by the US Department of Energy’s National Energy Technology Laboratory (NETL) for the LHSG (with a user specific number of 100 iterations).

Subsequently, the sampled material parameter sets are normalized, since the algorithms used for training the NN achieve better results with values between 0 and 1. The influence of the number of used input data is examined in Section 3.2.2. As already described in Section 2.1.1, the custom formulation for the yield function and the material parameter sets are used to determine value pairs (stresses vs. platic strain) in form of a table, which are then imported into the material card. With the help of the FE-model, described in Section 2.3, a high number of simulations with the respective material cards were executed. The automatically evaluated simulation results (FDC) with the corresponding material parameters formed the data basis for the training of the NN. The same abscissa positions (displacements) were evaluated for all data sets, to ensure the comparability of the data. Therefore, the distance between the evaluated points was reduced in the area with a tendency to higher curvature and in total 35 data points were used for evaluation and as input data set. Each input data set was labeled with the four parameters used for its calculation. Since results from FE simulations do not have random errors, the resulting data (e.g., force-displacement curves) are clean and noiseless. To make the NN robust to measurement errors, which always occurs in real experiments, a Gaussian noise with zero mean and variance $s^{2}$ is added to the resulting force data:

(5) $$F_{noise}=F\left(1+N\left(\mu =0,s^{2}=0.1\right)\right).$$

To better evaluate the performance of the NN and to detect possible overfitting, a train/validation data split is performed and additionally cross-validation (see Section 2.2) is used. Afterwards, a NN is set up and trained with the generated data by reversing the role of inputs and outputs. Since the prediction accuracy of a NN depends significantly on the HPs (e.g., number of neurons, activation function) chosen to create this model, an optimization of these HPs was performed in the following (see Section 2.2). In order to evaluate not only the deviation between given parameters and predicted parameters but also the more relevant difference between the resulting simulated FDC, these curves were calculated for each of the material parameter sets. For comparison, the same 35 evaluation points were used as before. After training the NN, it was applied to the experimentally determined input data (35 force values) to identify the corresponding set of material parameters. Again, the experimental test was simulated using the predicted material parameters respectively the generated material card and then the experimental and calculated FDC curves were compared. In the subsequent section, NN’s and the corresponding methods used in this paper are discussed in more detail.

### 2.2. Artificial Neural Networks

**Figure 3:**
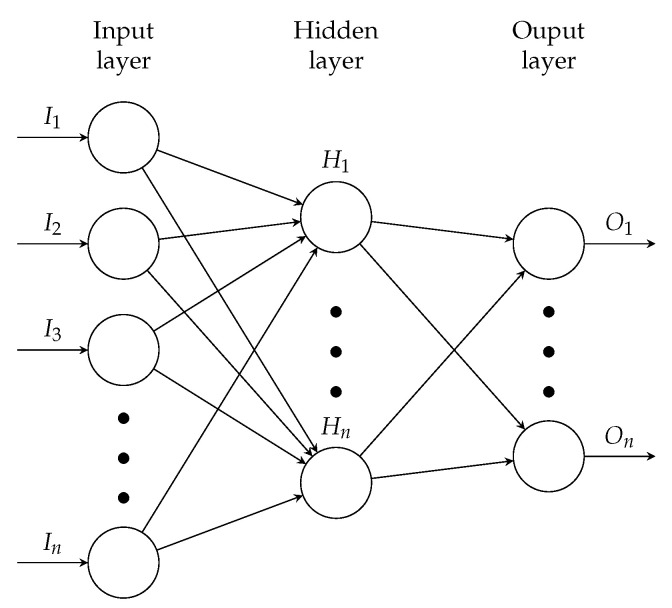
Schematic structure of a feedforward artificial neural network.

ANN (see Figure 3) are particularly effective in solving such problems where the correlations between the dependent and independent data are well known, but the detailed description with commonly used mathematical methods is too complex or even impossible [33,34]. In most cases, ANN are adaptive systems that adjust their structure based on external or internal information that flow through the network during the learning (training) phase [35]. During this learning process, the input data is mapped to the output data and the function used to do this is automatically adjusted repeatedly until a sufficiently accurate function is found to describe the input/output relationship. The trained ANN, in which the information gained from the learning process is available in the form of set weights and biases, can then be used to predict unknown relationships between input and output data [36]. In the present case, the ANN is a so-called feedforward artificial neural network. It consists of neurons organized into layers, where outputs from one layer are used as inputs into the following layer (see Figure 3).

The input layer (IL) is responsible for receiving information from the external environment. The hidden layer(s) are composed of neurons, which are responsible for extracting patterns associated with the process or system being analyzed. The output layer (OL) is also composed of neurons and thus is responsible for producing and presenting the final network outputs, which result from the processing performed by the neurons in the previous layers [35,36,37].

In analogy to the biological equivalent, activation functions in ANN’s represent the activation of a neuron. This activation function provides the input for each neuron by mapping the sum of the outputs of the previous layer accordingly. There are different formulations for the activation functions. One of the most frequently used is the *sigmoid* function:

(6) $$S\left(x\right)=\frac{1}{1+e^{-x}}.$$

The respective activation functions of the different layers have significant influence on the accuracy of the resulting prediction of the NN and thus represent a hyperparameter. HPs are settings that can be used to control or adjust the behavior of ML algorithms and are not adapted by the learning algorithm itself [38]. It has been shown that these parameters can have a significant effect on the prediction performance of an ML model [39]. The weights and the biases are the parameters of an FFANN to be determined during the training process. Further information can be found in [9,35,36,37,40].

There are only a few precise recommendations for the choice of ANN’s architecture in the literature, since this depends strongly on the present application case, which is rarely transferable. Generally, it was shown in [41,42] that ANN’s with any of a wide variety of large number of continuous non-linear HL activation functions and one HL with an arbitrary number of units is sufficient for the universal approximation property. Nevertheless, in this paper FFANN with 2 HL’s is also considered to investigate the influence. For more detailed information regarding the structure and architecture of ANN, please refer to [9,35,36,37,38,40].

Often, the general influences of HPs on model performance are known, but it is very complex to find the best set of interacting HPs. There are only a few recommendations or rules of thumb for the choice of ANN’s architecture and configuring their hyperparameters. To overcome this problem, there are approaches to objectively search different values for model HPs and choose a single set that results in a model, which achieves the best performance on a given dataset. These approaches are called HP tuning or HP optimization and a variety of different applicable libraries (e.g., *scikit-learn*, *Hyperas*) with different algorithms exist. For more detailed information on these, please refer to [28,38,43].

An hyperparameter search procedure involves the definition of a search space, which represents a volume to be searched, where each dimension represents a HP and the scale of the dimension are the values that the HP may take on (e.g., real-valued, integer-valued, categorical) [38,44].

In this paper, gridsearch (using *scikit-learn*), randomsearch (using *scikit-learn*) and HP optimization (using *Keras Tuner* and *Hyperas*) are applied to determine optimal HP sets. Gridsearch is the most basic strategy for the automated selection of HPs. Therefore, a search space is defined as a grid of HP values and each position in the grid is evaluated. An alternative to grid search is the random search algorithm, where a search space as a bounded domain of HP values and randomly sample points with the possibility of a specified distribution in that domain is defined. However, the search for appropriate HPs can be formulated as an optimization problem. In this case, the decision variables are the HPs and the cost to be optimized is the validation set error that results from training using these parameters. In this paper, the two libraries *Keras Tuner* (V.1.0.1) [45] with a *Hyperband* as well as a Bayesian optimization algorithm and *Hyperas* (V.0.4.1) with a TPE algorithm are used. The Bayesian optimization provides a principled technique based on Bayes Theorem to direct a search of a global optimization problem that is efficient and effective. Therefore, a probabilistic model of the objective function is built, called the surrogate function, and then it is searched efficiently with an acquisition function before candidate samples are chosen for evaluation on the real objective function [44]. In contrast, Hyperband is a bandit-based strategy for HP optimization that iteratively allocates resources to a set of random configurations [46]. The Tree-of-Parzen-Estimators (TPE) is a sequential model-based optimization (SMBO) approach, which construct models to approximate the performance of HPs based on historical measurements, and then subsequently chooses new HPs to test, based on this model [47]. For more detailed information on the used optimization algorithms refer to [38,44,45,46,47,48].

The hyperparameters investigated in this paper are listed in the Appendix A in Table A3 for a defined default NN. Besides the possibility to use different combinations of these HPs, it is also possible to use different sets of hyperparameters (e.g., different activation functions for the used layers), which further increases the number of possible HP combinations [35,36,38,49,50]. It should be mentioned that there are other important HPs, such as the learning rate or the momentum. Their consideration would go beyond the scope of this work, but they should be considered in further studies.

Since in this present case the output is a continuous quantity and not a label of discrete classes, it can be considered to be regression problem. The goal of the training process is to find a model $M^{INV}$ that matches the examples at best. The cost function quantifies the error between the predicted output and the labeled output and presents it in form of a single real number. A commonly used function, which is also applied in this case is the mean squared error (MSE), which tries to minimize the average error between all ANN’s outputs and all labeled outputs over all data sets. The minimization of this cost function using a gradient descent (GD) optimization for the FFANN is called backpropagation algorithm. Using the backpropagation algorithm, the synaptic weights and biases are automatically adjusted in several iterations, successively reducing the error produced by the ANN. There are different minimization algorithms, but in this paper we limited ourselves to the use of the backpropagation algorithm with GD optimization. Furthermore, there are different GD optimization algorithms, whereby some of them are tested in this paper (see Section 3.2.6) [34,35,36,43].

To better evaluate the performance of ML models, e.g., regarding unseen data, the existing data is divided into training and validation data sets. To estimate the performance even better, the cross-validation method is often used, especially when there is a limited amount of data available. The (k-fold-)cross-validation procedure has a single parameter called k that refers to the number of non-overlapping groups that a given data sample is to be split into. A total of k models are fit and evaluated on a hold-out validation sets and the mean performance is reported. Further information can be found in [35,38].

### 2.3. Data Generation by Numerical Simulations

**Figure 4:**
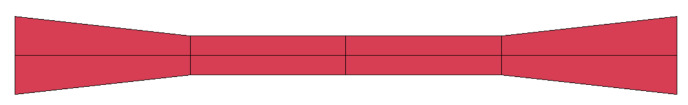
FE discretization of the tensile specimen.

As explained in Section 2.1.2, the data required for the training of the NN are generated by numerical simulations. The final prediction with the trained NN is performed using the force data of the experimentally tested specimens. For both material PI methods, FE simulations were carried out using a finite (shell-)element discretization and the material model *MAT024* (*MAT_PIECEWISE_LINEAR_PLASTICITY*) with the custom formulation for the yield function 1. Apart from the four material parameters, which were to be identified by the method applied, the following material parameters were used: E=2127.3 MPa, ν=0.35, ρ=1040$\frac{\mathrm{kg}}{{\mathrm{mm}}^{3}}$, $fail=1*10^{21}$ (plastic strain to failure—default value). Strain rate dependence, anisotropy, failure and other phenomena were not considered. In order to reduce the time required for the simulations, the geometry of the tensile specimen is discretized with only 8 elements (see Figure 4). To ensure this procedure, additional FE simulations with a higher number of elements were carried out, which provided comparable results. For performance reasons, all tensile tests were calculated in a single simulation run, since for these small FE-models the initialization time for *LS-DYNA* is larger than the computing time. The input file for the solver was created automatically by custom scripts using includes.

The simulation was performed displacement controlled and the resulting outputs (force/time and displacement/time) were written to *secforc* text files and *nodout* text files, respectively. As described in Section 2.1.2, 35 points at the same abscissa position with higher distribution density to the non-linear part of the curve were automatically evaluated with the help of created Python scripts.

### 2.4. Additive Manufacturing and Mechanical Characterization of Test Specimens—Experimental Set-Up

Mechanical characterization of the additively manufactured specimens was conducted according to DIN EN ISO 527, using test specimen type 1A. For manufacturing of the specimens, the pro-consumer machine *X400* by German *RepRap GmbH* (Feldkirchen, Germany) and ABS from *Innofil3D B.V.* (Emmen, The Netherlands) was selected. Before printing, the ABS were dried at 60 ${}^{\circ }$C for 4 h. The environmental conditions was constant with an ambient temperature of about 22 ${}^{\circ }$C and a relative humidity of 40–45%. Table 1 shows the used process parameters for material extrusion process. All five test specimens were manufactured in a single build process.

**Figure 5:**
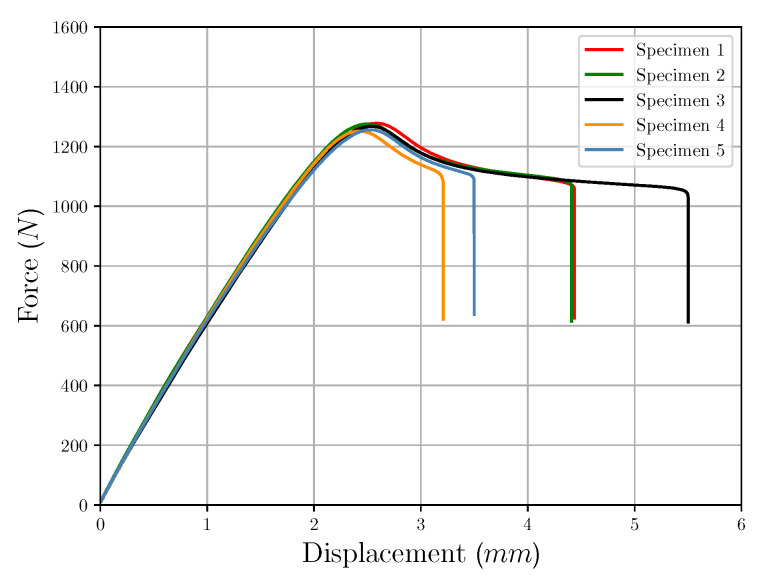
Experimental results.

The experimental testing was done by the universal testing system *Instron 5966* (*Instron^®^GmbH*, Darmstadt, Germany) with a 10 kN load cell and using an extensometer for strain measurement. The testing speed was set to 1 mm per minute. Figure 5 shows the stress-strain curves of the additively manufactured test specimens. Both Young’s modulus (2055.33 MPa) and maximum tensile stress (30.70 MPa) exhibit rather low standard deviations 155.94 MPa and 0.64 MPa, respectively, while elongation at break shows lager scattering. Thus, the experimental data are well applicable for the investigations in this paper, since the elongation at break will not be considered at first. Higher strains would lead to difficulties in the numerical simulation, especially in combination with the custom yield curve. Since this is not the focus of the investigation and the custom formulation of the yield curve only serves to describe the existing material behavior without damage and other effects, the experimental results of test specimen 5 were selected as data basis for further investigations. This sample shows the representative material behavior, but has a lower elongation at break than most other samples.

## 3. Results and Discussion

### 3.1. Iterative Optimization Results

**Figure 6:**
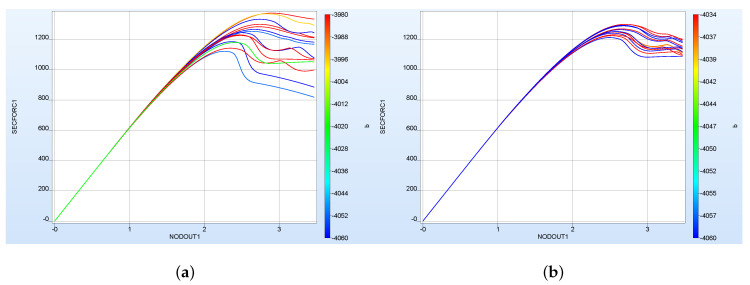
FDC from the parameter identification process with *LS-OPT* at (**a**) iteration 1 and (**b**) iteration 4 (*SECFORC1*—*LS-DYNA* output for the force and *NODOUT1*—for displacement).

Out of the maximum 16 possible iterations, 9 iterations with 14 sample simulations each were needed to find suitable material parameters. Figure 6 shows exemplarily the results from *LS-OPT* at iteration 1 and iteration 4. Table 2 shows the determined material parameters and the MSE resulting from the comparison to the experimental force-displacement curve.

The force-displacement curve obtained with the identified material parameters is shown in Section 3.3. and is compared with the results from the NN-based method.

### 3.2. FFANN Results

A systematic experimental scheme was developed (see Appendix A Table A1) for the quantitative and qualitative evaluation of different boundary conditions/settings—e.g., amount of training data and NN hyperparameters—based on the prediction accuracy of the NN direct inverse identification method. In this scheme, the boundary conditions to be investigated were systematically varied in so-called *Runs* and thus their influence on the prediction accuracy was evaluated. Among others, the material parameter range used for the training of the NN was varied. The ranges used can be found in the Appendix A in Table A2. A default set (see Appendix A Table A3) was used to ensure comparability. The settings resulting from the HP optimization are listed in the Appendix A in Table A7.

For evaluation purposes, both the MSE between labeled and predicted material parameters (a, b, c, d) of the validation set and the MSE between the resulting FDC are listed in Table A1. Additionally, using the experimental data as input, material parameters were predicted by the NN and subsequently respective FDC were calculated by numerical simulations. The MSE between experimental and calculated FDC is also shown in this table.

In the following, from Section 3.2.1, Section 3.2.2, Section 3.2.3, Section 3.2.4, Section 3.2.5 and Section 3.2.6. the results of the corresponding boundary conditions/settings are presented and evaluated. In Section 3.3. the resulting FDC of selected *Runs* are presented and compared with the FDC of the iterative optimization approach (with *LS-OPT*) as well as the experimental curve.

#### 3.2.1. Sampling Strategies

**Figure 7:**
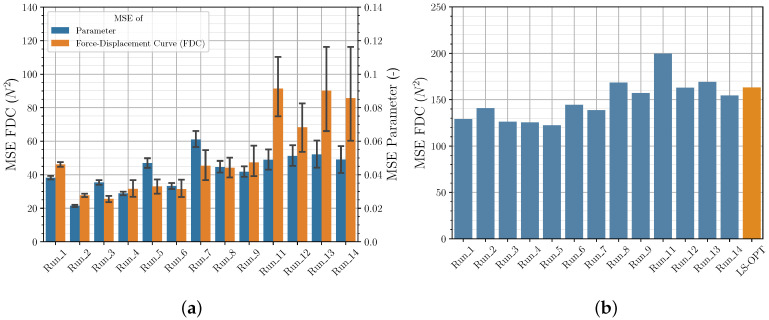
Sampling strategy and quantity of input data—comparison of MSE of material parameter and FDC from (**a**) validation data and (**b**) experimental data.

Figure 7a shows that in general LHS leads to better (lower) results for both the MSE of parameters and the FDC of the validation data set than FFA (in all bar charts a 95% confidence interval is shown). This was to be expected, since using the same number of samples, LHS provides a wider distribution and thus more information about the investigated parameter space than FFA. However, it should be mentioned that this observation did not occur in preliminary studies (not presented here). In these fewer neurons were used, and fewer epochs were trained. This suggests that the additional information provided by LHS about the input/output relationship also requires a higher complexity of the NN as well as longer training to be detectable. This once again illustrates the need to investigate the correlations to achieve greater prediction accuracy.

However, Figure 7b shows that the better performance of the LHC in contrast to the FFA is not represented in the FDC of the experimental data set. Furthermore, as expected, the results of the investigation show that with a lower number of sampling points, the importance of a better distribution of the sample points increases. Due to high computational costs, LHSG was only used for a smaller number of sampling points. Contrary to LHS, LHSG showed a tendency to provide better results also for the FDC MSE for the experimental data set, but this should be investigated in further studies.

**Figure 8:**
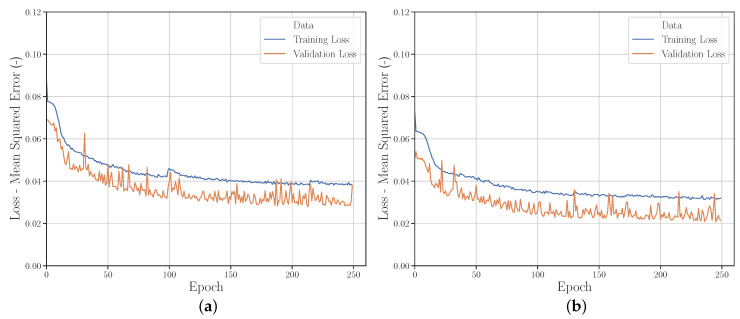
Learning curves from (**a**) *Run_1* and (**b**) *Run_2*.

Figure 8 shows the two learning curves for *Run_1* (FFA) and *Run_2* (LHS). Both sampling strategies show a similar behavior. The learning curves of the training data have a lower variance for both *Runs*. In contrast, the learning curves of the validation data show a lower MSE on average (this is explained in more detail in Section 3.2.4).

#### 3.2.2. Quantity of Input Data

Figure 7a,b show that for a training set size of 10,000 and 4096 parameter sets there are no significant differences in the MSE examined. This applies to both the MSE of the validation data and the MSE of the experimental data. Only from a used train set size of 2401 the MSE of the parameters using the FFA increases as expected. If the train set size is further reduced, the MSE of the validation data and the experimental data increases significantly for all sampling strategies used. This is more evident for the FFA used than for LHS and LHSG (*Run_10* with FFA not shown). For the investigated use case it can therefore be stated that the training set size of approx. 4000 parameter sets is sufficient and that the prediction performance of the default NN cannot be further significantly increased by a higher number of samples.

#### 3.2.3. Input Data Range

**Figure 9:**
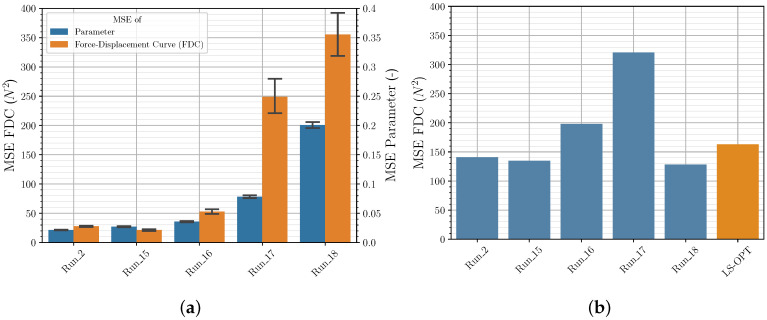
Input data ranges comparison of MSE of material parameter and FDC from (**a**) validation data and (**b**) experimental data.

Another boundary condition that was investigated is the material parameter range, on which the NN was trained (see Appendix A Table A2). The initial range (referred to as range 1) of the four given material parameters were reduced by 10, 30 and 50% (referred to as range 2, 3 and 4) to quantify the influence. To test how well the NN can determine parameters that lay outside the training range and how this is being reflected in the FDC, the NN was trained at range 1 and validated at range 1, 2, 3 and 4. The results are shown in Figure 9, with *Run_2* (training range 1 and validation on range 2) for comparison. The prediction accuracy varies only slightly for both the validation data set and the experimental data set if the same training range and either validation range 1 or range 2 are used for both *Runs*. However, if the training range is reduced and the validation range is maintained, the MSE of the parameters and the FDC will, as expected, increase significantly. One outlier is *Run_18*, which shows a very low MSE of the FDC of the experimental data set. It is assumed that the weights and biases were randomly optimized during training in such a way that these specific experimental data can be described particularly well with the calculated FDC. However, it is shown that the choice of the parameter range is essential for the sampling and the subsequent training and should be chosen in such a way that the behavior of the material to be simulated can be described with parameters within the training range.

#### 3.2.4. Validation Set Sizes

**Figure 10:**
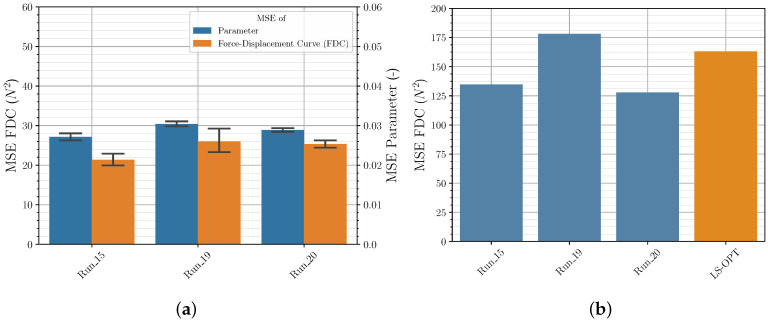
Comparison of MSE of material parameter and FDC from (**a**) validation data and (**b**) experimental data with different validation set sizes.

In addition to the parameter range, the training/validation data ratio was investigated (see Figure 10). For this purpose, the validation set size was incrementally increased from 2401 sets to 10,000 sets (50%/50% training/validation ratio). For the MSE of the parameters and the FDC no significant difference can be seen. However, the variance of the MSE of the parameters of the validation set decreases significantly with increasing size of the validation set. It is assumed that the NN prediction performance can be better evaluated with a more even training/validation ratio.

**Figure 11:**
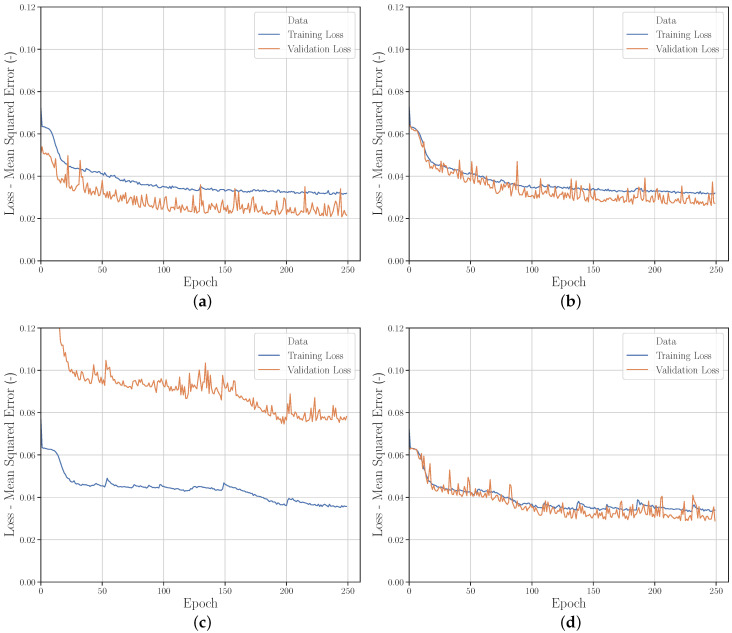
Learning curves for different variations of parameter ranges and training set/validation set ratio; (**a**) *Run_2*, (**b**) *Run_15*, (**c**) *Run_17* and (**d**) *Run_20*.

The comparison of the learning curves Figure 11a,d shows that the prediction performance of the NN on the validation set at *Run_20* corresponds much more to the prediction performance of the NN on the training set than at *Run_2*. The validation set is therefore more representative and therefore better suited for evaluation. The learning curves of *Run_15* and *Run_17* (Figure 11b,c) show that this can also be achieved by varying the training and validation range. If the validation range is located outside the training range, it is rather inappropriate for evaluating the performance of the NN. Thus, the studies indicate that a training/validation ratio of approx. 66%/33%–50%/50% should be chosen for the highest possible prediction accuracy and evaluability of the NN performance.

#### 3.2.5. Data Modification with Gaussian Noise

**Figure 12:**
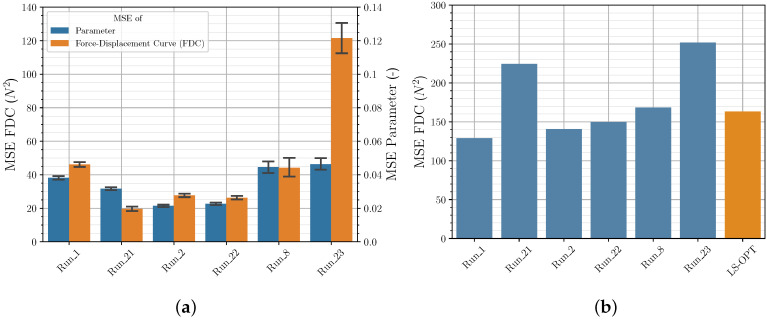
Comparison of MSE of material parameter and FDC from (**a**) validation data and (**b**) experimental data with noisy training data.

As mentioned above, the influence of the application of a gaussian noise on the force values of the training data was investigated. The aim was to increase the prediction accuracy of the NN or to increase its ability to generalize (performance on unknown error-caused data). Therefore, the results were examined with and without noise for the three different sampling strategies (see Figure 12). Exclusively for the MSE of the parameters and the FDC of the validation set a small improvement was observed. Contrary to expectations, all other results of both the MSE of the validation set and the experimental set remained unchanged or even became worse. This should be investigated in further work and possibly other standard deviations for the gaussian noise should be used.

#### 3.2.6. Hyperparameters

To evaluate the influence of different HPs on the present application and to compare the search methods, trainings with different HP settings were conducted under constant boundary conditions/settings. The MSE of the parameters of the training sets was then evaluated.

#### Gridsearch

For the gridsearch method, in each case one hyperparameter was varied in different steps (see Appendix A Table A4)—vertical run and a cross-validation with three folds was used. The HPs not varied per run correspond to the default set (see Appendix A Table A3). All other boundary conditions/settings correspond to *Run_2* (see Appendix A Table A1). The gridsearch results are listed in the Appendix A in Table A5, where the worst/best result of a HP are highlighted red/green, respectively. The kernel initializer, the activation function, the dropout value and the selected GD optimizer showed a particularly high influence on the performance of the NN.

**Figure 13:**
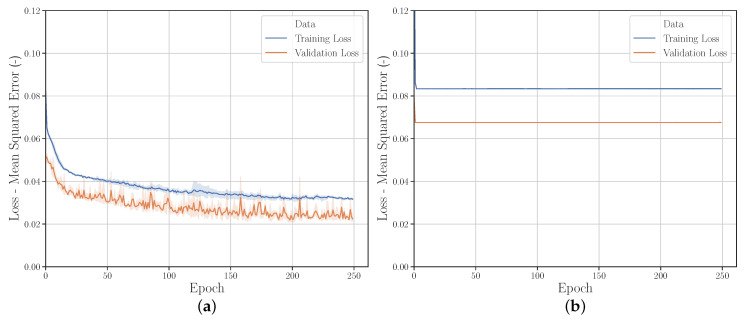
Learning curves with kernel initializer 1 (**a**) *HE Normal* and (**b**) *Zero*.

**Figure 14:**
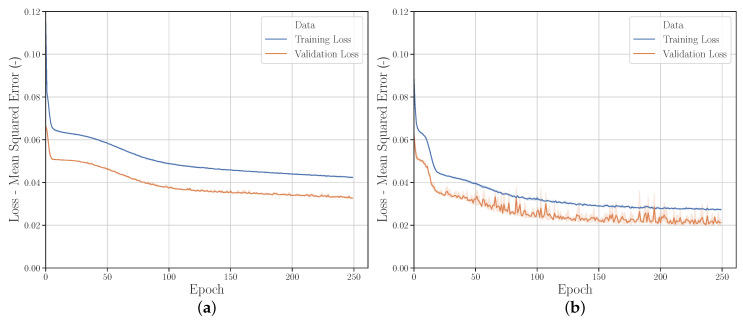
Learning curves with activation (**a**) *Softmax* and (**b**) *Hard Sigmoid*.

**Figure 15:**
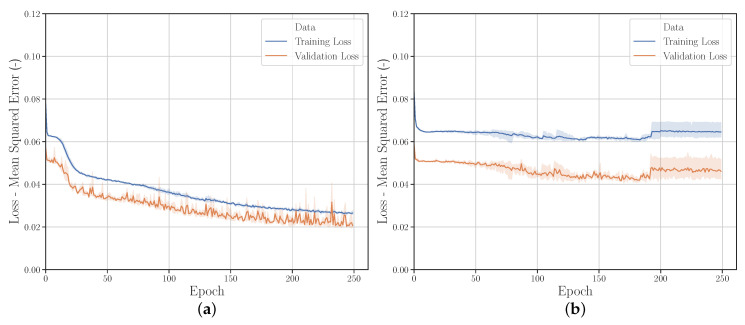
Learning curves with dropout (**a**) 0.00 and (**b**) 0.40.

**Figure A1:**
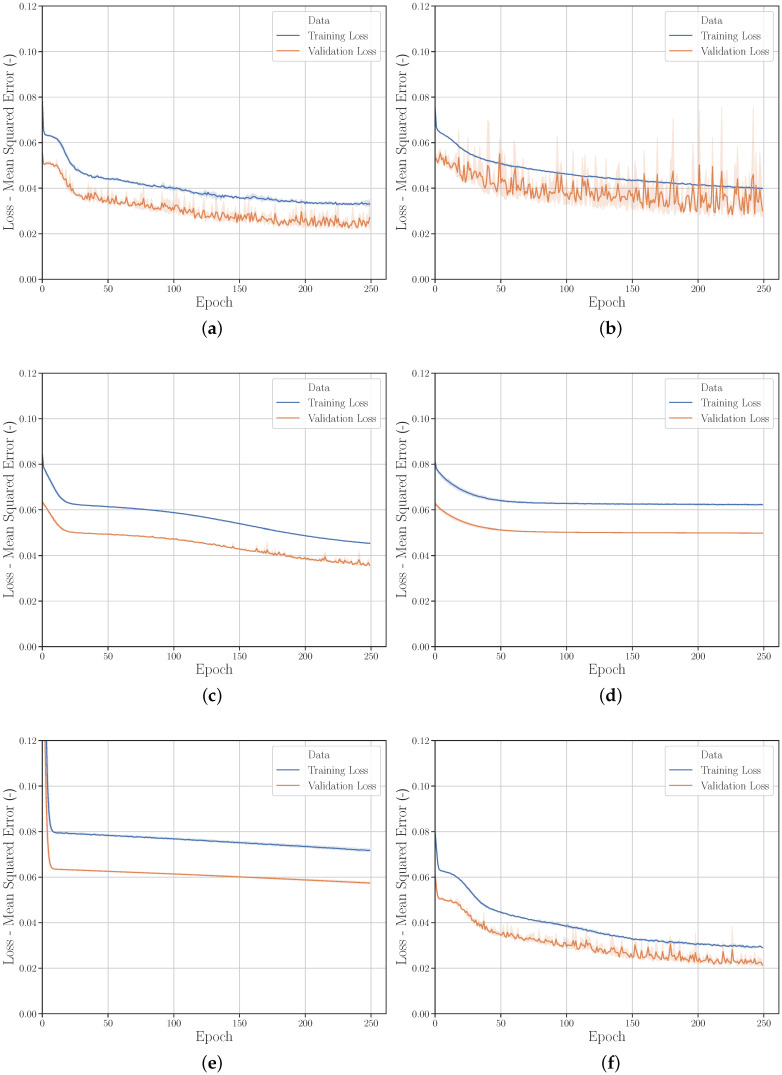
Learning curves with optimizer (**a**) *Adam* and (**b**) *Rmsprop*, (**c**) *SGD*, (**d**) *Adagrad*, (**e**) *Adadelta* and (**f**) *Adamax*.

Figure 13, Figure 14 and Figure 15 and Figure A1 show a selection of learning curves for varying HP settings. Figure 13b illustrates the low performance of the kernel initializer *Zero* compared to *HE Normal*.

Figure 14 shows that although the variance of the learning curve can be greatly reduced by using the activation function *SoftMax*, the prediction accuracy is significantly lower compared to *Hard Sigmoid*.

Figure 15b shows that when using a higher dropout value (0.40) the prediction accuracy of the NN decreases and from approx. training epoch 190 the variance increases in steps.

Figure A1 in the Appendix A shows the learning curves of selected GD optimizers. It can be seen that the progression of the NN training differs significantly for different GD optimizers. The learning curve of the validation set with *Rmsprop* (Figure A1b) shows a very high variance compared to *SGD*, *Adagrad* and *Adadelta* (c, d, e), whereas with these the prediction accuracy is significantly worse. *Adamax* showed the best performance (Figure A1e).

#### Randomsearch

As previously explained in Section 2.2 there are cross effects between the individual HPs, which can have a significant influence on the performance of the NN. In order to better evaluate the HP settings of the NN regarding the present use case, a random search with 100 variants on the same search range as the grid search (see Appendix A Table A4) and with 3-fold cross-validation was carried out. However, a random variant was chosen for each HP. The best and worst five results including the randomly selected HP are listed in the Appendix A in Table A6. Similar to the results of the gridsearch the NN achieved very good results with the GD optimizer *Adamax*, whereas the results with *Adadelta* were rather worse. However, the best results of the gridsearch could not be achieved by randomsearch. This is probably due to the small number of 100 variants examined, which should be increased for future investigations. In this case, cross effects should continue to be investigated systematically with the help of a statistical sensitivity analysis.

#### Hyperparameter Optimization

Using different HP optimization algorithms of the libraries *Keras Tuner* and *Hyperas* HP settings were determined based on the boundary conditions/settings of *Run_1* and *Run_2*. Contrary to the approach with gridsearch and randomsearch, the use of a second HL was additionally made possible, which in turn also represents a HP. As HP search range identical settings as for the search variants (Appendix A Table A4) were used, but with the following differences:

- Epochs per default 250
- Neurons from 50 to 500 in steps of 25
- GD optimizers without SGD

**Figure 16:**
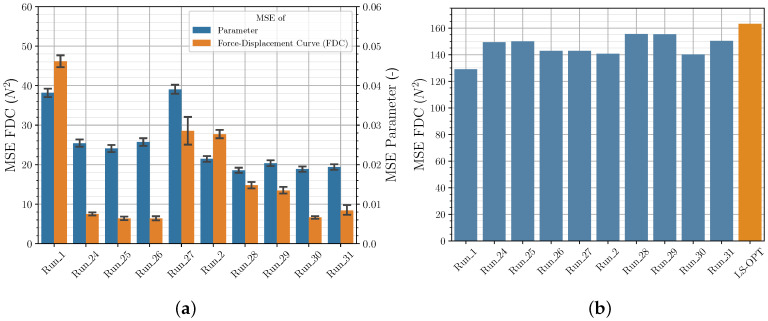
Comparison of MSE of FDC from (**a**) validation data and (**b**) experimental data for different NN from HP optimization.

The default settings as well as the NN settings determined for the *Runs* with varying NN are listed in the Appendix A in Table A7. Figure 16 shows the results of the *Runs* with the optimized NN hyperparameters and for comparison purposes *Run_1* and *Run_2*. It can be seen that all HP optimizations lead to NN settings, which result in a significantly better MSE of the parameters and the FDC of the validation set. Especially the MSE of the FDC of the validation set could be reduced significantly. The Bayesian optimization with the *Keras Tuner* library using the LHS shows the best results. When looking at the MSE of the FDC of the experimental data set (Figure 16b), it is noticeable that the NN of the HP optimization achieves consistently good results compared to the *LS-OPT* solution, but the significant reduction of the MSE of the validation set is not reflected. This cannot be explained at this point in time and needs further investigation, as the main goal is to maximize the performance of the NN for experimental data sets. The data of the validation set may not be representative enough or the material model with the selected yield curve may not be sufficiently suitable to increase the performance with respect to the experimental data set.

### 3.3. Comparison of Iterative and Direct Inverse Procedures

**Figure 17:**
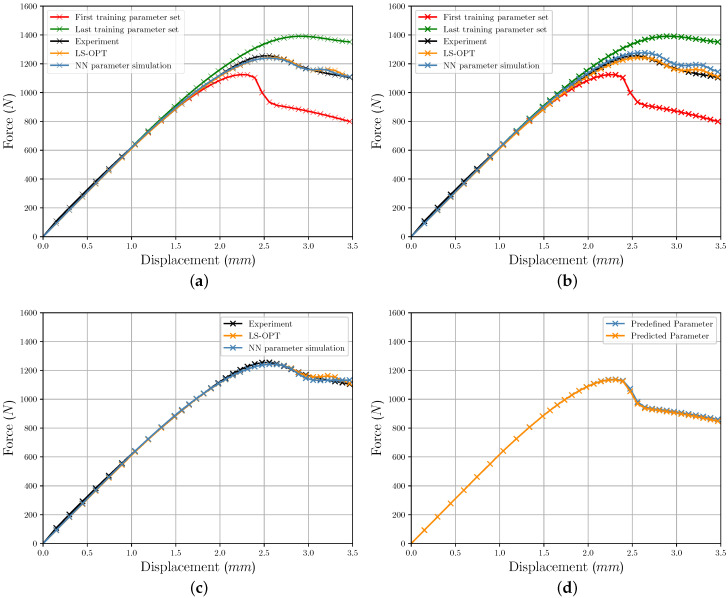
Comparison of calculated FDC with parameters from iterative and NN-based process for (**a**) *Run_5*, (**b**) *Run_10*, (**c**) *Run_31* and (**d**) for one randomly chosen labeled and the corresponding predicted parameter set of *Run_26*.

Figure 17a–c show selected FDC resulting from FE simulations with the identified parameters using NN and *LS-OPT*. For comparison purposes, the experimentally determined FDC is shown. The custom formulation of the yield curve in combination with the chosen material model *MAT024* leads in some cases to an unexplainable further peak of the FDC after reaching the maximum force. The table of the plastic strains and associated stresses, which was generated with the help of the custom yield curve approach, does not show this peak. The reason could not be finally clarified. However, it should be mentioned again that this peak results from the FE simulation and is not a specific weakness of the applied NN-based method. Nevertheless, it is possible to evaluate the NN-based PI method and to verify its applicability as well as to compare the two parameter identification methods.

Figure 17a,b additionally shows the FDCs determined with the first and last training parameter set and illustrating the parameter range. Figure 17a shows that the FDC of both methods produce very similar results, although the NN-based method achieves slightly better results for the simulation of the experimental tensile test. Figure 17b displays the FDC at high MSE of the determined parameters. Figure 17c shows the resulting FDC from the NN parameter prediction with previous HP optimization. This also leads to good results and does not exhibit the second peak after the maximum force. Figure 17d shows an example of the FDC calculated with predefined (used for NN training) and predicted parameter, which results in very good agreement. Summarizing, it can be said that the NN-based method is suitable for identifying material parameters for the present application. When using suitable boundary conditions/settings as well as HPs for the NN, even better results could be determined by the NN-based method compared to the commonly used iterative optimization method (compare Table 2 and Table A1). In the reduced results in Table 3 it can be seen that neither the *Runs* with the lowest MSE of the parameters nor the FDC lead to the lowest MSE of the FDC of the experimental data set. This indicates that the increase in prediction accuracy through optimization is not directly reflected in the experimental data set.

It is conceivable that the training and test set may not be sufficiently representative to provide the best possible results for the experimental data set, or the NN trained for *MAT024* is not able to generalize sufficiently well. As this phenomenon has not yet been fully clarified, it should be investigated further. This is of major relevance as the overall goal is to configure and train the NN to perform best on unknown experimental data.

## 4. Conclusions and Outlook

In this paper, a method was presented to identify material parameters of the yield curve using a FFANN for the numerical simulation of additively manufactured test specimen under uniaxial tensile load. The labeled data required for the training of the FFANN were generated by numerical simulations with *LS-DYNA* and using *MAT024*. In order to describe the elastic-plastic material behavior of the ABS without having to consider more complex mechanisms such as damage, for which a PI would be required as well, a custom formulation for the yield curve consisting of a second-order polynomial and a root function was created.

The resulting material parameters as well as the results of the following structural simulation of the iterative optimization-based and the direct inverse NN-based method for PI were compared. The results prove the suitability of the NN for the identification of the yield curve parameter of the investigated ABS. Using suitable boundary conditions and settings, the NN-based method achieved better results than the commonly used iterative optimization method. The results confirm the authors assumption that such feedforward neural networks could be used in the future to perform parameter identifications of the complex property spectrum for complete material models of thermoplastics. This would have the advantage that the necessary experimental investigations for the PI of a material could be reduced to a minimum, because the FFANN can be trained with the help of data from numerical simulations. An even greater advantage is the reusability of the trained FFANN for similar materials as well as the corresponding PI within seconds. Thus, product development times could be reduced.

In contrast, if the material behavior differs only slightly from the conventional used iterative optimization approach, computationally intensive numerical simulations would be required. A prerequisite for the repeated use of a trained FFANN, however, would be that the specific material behavior of the material to be characterized is represented within the input data used for the training. As the PI process only requires expert knowledge for the creation of the FFANN, but not for the actual identification process, the results would be objectively comparable. Thus, especially in early development phases, the influences of different materials (with similar material characteristics) on the structural-mechanical properties of developed components could be evaluated and compared. Conceivable would be a polymer that shows similar material-mechanical behavior, e.g., ±30% maximum tensile strength or a different contour of the non-linear stress-strain curve.

Essential for the accuracy of the predicted parameters and thus the success of the method is the choice and processing of the training data of the FFANN as well as the choice of the hyperparameters, as presented in this paper. The investigations should facilitate and reduce the choice of boundary conditions and settings for future work in this field of work. Furthermore, different methods for the selection and optimization of the HPs of the FFANN were shown and compared. The highest increase of the prediction accuracy of the validation set could be achieved by HP optimization with the library *Keras Tuner* and the Bayesian optimization algorithm. However, this increase in prediction accuracy was not directly reflected in a higher accuracy of the MSE of the experimental data set, which should be investigated in future work. Furthermore, a reduction of the MSE of the parameters was usually, but not always accompanied by a reduction of the MSE of the FDC, which should also be examined in further studies.

In addition, it is planned to extend the spectrum of properties of thermoplastics to be investigated in subsequent work to finally achieve an FFANN with which parameters for more complex material models can be determined and thus to be able to represent the extensive material behavior of thermoplastics. Thus, it would have to be evaluated whether and with which accuracy parameters for strain rate dependence, damage, failure or other material characteristics can be determined in addition to the material parameters for the yield curve. For this purpose it requires investigations whether and how different NN’s for the material characteristics must interact with each other. 

## Figures and Tables

**Table 1:** Used process parameters for the manufacturing of the test specimens.

Material	Color	Extrusion Speed	Build Platform Temperature	Nozzle Temperature	Layer Thickness	Raster Angle	Perimeter Shells
ABS	black	40 mm/s	100 ${}^{\circ }$C	245 ${}^{\circ }$C	0.2 mm	±45 ${}^{\circ }$C	2

**Table 2:** Identified material parameters using *LS-OPT* for the simulation of the experimental specimen and the corresponding FDC MSE.

Parameter *a*	Parameter *b*	Parameter *c*	Parameter *d*	MSE FDC
52,514	−4056.63	11.231	539.29	163.14 $N^{2}$

**Table 3:** Reduced settings and results of NN-based material parameter identification *Runs*.

Run	Mean MSE Parameter Val. Set (−)	Mean MSE FDC Val. Set ($N^{2}$)	MSE FDC Exp. ($N^{2}$)
5	0.0469	32.9	122.4
25	0.0241	6.4	150.1
26	0.0257	6.4	142.9

## Abbreviations

The following abbreviations are used in this manuscript:

ABS	Acrylonitrile butadiene styrene
AM	Additive Manufacturing
ANN	Artificial Neural Network
EXP	Experiment
FE	Finite Element
FFA	Full Factorial Approach
FFANN	Feedforward Artificial Neural Network
GD	Gradient Descent
HL	Hidden Layer
HP	Hyperparameter
IL	Input Layer
LHS	Latin Hypercube Sampling
LHSG	Latin Hypercube Sampling with genetic space filling
ML	Machine Learning
NN	Neural Network
OL	Output Layer
PI	Parameter Identification
SGD	Stochastic gradient descent
TPE	Tree-of-Parzen-Estimators

## Appendix A

**Table A1:** Settings and results of NN-based material parameter identification *Runs*.

Run	Sampling Strategy	Train Set Size	Val. Set Size	Train Range	Val. Range	NN	Noisy Data	Mean MSE Parameter Val. Set (−)	Mean MSE FDC Val. Set ($N^{2}$)	MSE FDC Exp. ($N^{2}$)
**1**	FFA	10,000	2401	1	2	Default	No	0.0382	46.2	129.0
**2**	LHS	10,000	2401	1	2	Default	No	0.0215	27.7	140.7
**3**	FFA	4096	1296	1	2	Default	No	0.0354	25.5	126.1
**4**	LHS	4096	1296	1	2	Default	No	0.0288	31.4	125.5
**5**	FFA	2401	625	1	2	Default	No	0.0469	32.9	122.4
**6**	LHS	2401	625	1	2	Default	No	0.0332	31.4	144.4
**7**	FFA	625	256	1	2	Default	No	0.0609	45.3	138.6
**8**	LHS	625	256	1	2	Default	No	0.0445	44.1	168.3
**9**	LHSG	625	256	1	2	Default	No	0.0417	47.3	157.1
**10**	FFA	256	81	1	2	Default	No	0.0972	626.7	780.5
**11**	LHS	256	81	1	2	Default	No	0.0489	91.4	199.5
**12**	LHSG	256	81	1	2	Default	No	0.0512	68.2	162.8
**13**	LHS	150	50	1	2	Default	No	0.0521	90.1	169.1
**14**	LHSG	150	50	1	2	Default	No	0.0491	85.7	154.4
**15**	LHS	10,000	2401	1	1	Default	No	0.0271	21.3	134.8
**16**	LHS	10,000	2401	2	1	Default	No	0.0357	52.7	198.0
**17**	LHS	10,000	2401	3	1	Default	No	0.0782	248.9	320.6
**18**	LHS	10,000	2401	4	1	Default	No	0.2009	355.5	128.2
**19**	LHS	10,000	5000	1	1	Default	No	0.0304	26.0	178.2
**20**	LHS	10,000	10,000	1	1	Default	No	0.0289	25.3	127.9
**21**	FFA	10,000	2401	1	2	Default	Yes	0.0317	19.7	224.4
**22**	LHS	10,000	2401	1	2	Default	Yes	0.0227	26.3	149.7
**23**	LHS	625	256	1	2	Default	Yes	0.0463	121.6	251.9
**24**	FFA	10,000	2401	1	2	HPO1	No	0.0254	7.5	149.3
**25**	FFA	10,000	2401	1	2	HPO2	No	0.0241	6.4	150.1
**26**	FFA	10,000	2401	1	2	HPO3	No	0.0257	6.4	142.9
**27**	FFA	10,000	2401	1	2	HPO4	No	0.0390	28.5	142.9
**28**	LHS	10,000	2401	1	2	HPO5	No	0.0185	14.8	155.5
**29**	LHS	10,000	2401	1	2	HPO6	No	0.0204	13.5	155.3
**30**	LHS	10,000	2401	1	2	HPO7	No	0.0188	6.6	140.2
**31**	LHS	10,000	2401	1	2	HPO8	No	0.0194	8.4	150.3

**Table A2:** Defined material parameter ranges.

Parameter Range	$a_{\mathrm{Min}}$	$a_{\mathrm{Max}}$	$b_{\mathrm{Min}}$	$b_{\mathrm{Max}}$	$c_{\mathrm{Min}}$	$c_{\mathrm{Max}}$	$d_{\mathrm{Min}}$	$d_{\mathrm{Max}}$
1	50,000	54,000	−4060	−3980	10.500	12.800	510.00	555.00
2	50,200	53,800	−4056	−3984	10.615	12.685	512.25	552.75
3	50,600	53,400	−4048	−3992	10.845	12.455	516.75	548.25
4	51,000	53,000	−4040	−4000	11.075	12.225	521.25	543.75

**Table A3:** Default neural net setting.

(Hyper-)Parameter	Setting
Batch Size	12
Epochs	250
Neurons (Input Layer)	35
Hidden Layers	1
Neurons	250
Kernel Initializer (HL)	Normal
Activation (HL)	Relu
Dropout (HL)	0.05
Neurons (Output Layer)	4
Kernel Initializer (OL)	Normal
Activation (OL)	4
Neurons (Output Layer)	Linear
Gradient Descent Optimizer	Adam

**Table A4:** Gridsearch/Randomsearch search range (* = default parameter).

N	Batch Size	Epochs	Neurons (HL)	Kernel Initializer (HL)	Activation (HL)	Dropout (HL)	Kernel Initializer (OL)	Optimizer
**1**	3	75	25	Normal *	Softmax	0.00	Normal *	Adam *
**2**	6	100	50	Uniform	Softplus	0.05 *	Uniform	Rmsprop
**3**	9	125	75	Glorot Uniform	Softsign	0.10	Glorot Uniform	SGD
**4**	12 *	150	100	Lecun Uniform	Relu *	0.15	Lecun Uniform	Adagrad
**5**	15	175	125	Zero	Tanh	0.20	Zero	Adadelta
**6**	18	200	150	Glorot Normal	Sigmoid	0.25	Glorot Normal	Adamax
**7**	21	225	175	He Normal	Hard Sigmoid	0.30	He Normal	Nadam
**8**	24	250 *	200	He Uniform	Linear	0.35	He Uniform	−
**9**	27	−	225	−	Selu	0.40	−	−
**10**	30	−	250 *	−	Elu	0.45	−	−
**11**	33	−	275	−	Exponential	0.50	−	−
**12**	36	−	300	−	−	−	−	−
**13**	39	−	325	−	−	−	−	−
**14**	−	−	350	−	−	−	−	−
**15**	−	−	375	−	−	−	−	−
**16**	−	−	400	−	−	−	−	−
**17**	−	−	425	−	−	−	−	−
**18**	−	−	450	−	−	−	−	−
**19**	−	−	475	−	−	−	−	−
**20**	−	−	500	−	−	−	−	−

**Table A5:** Gridsearch training results—training MSE of material parameters.

N	Batch Size	Epochs	Neurons (HL)	Kernel Initializer (HL)	Activation (HL)	Dropout (HL)	Kernel Initializer (OL)	GD Optimizer
**1**	0.0380	0.0402	0.0323	0.0304	0.0406	0.0253	0.0322	0.0329
**2**	0.0328	0.0393	0.0320	0.0310	0.0375	0.0321	0.0324	0.0364
**3**	0.0343	0.0379	0.0329	0.0324	0.0301	0.0338	0.0312	0.0447
**4**	0.0308	0.0350	0.0341	0.0333	0.0314	0.0426	0.0332	0.0616
**5**	0.0317	0.0303	0.0291	0.0834	0.0389	0.0438	0.0318	0.0708
**6**	0.0288	0.0327	0.0312	0.0301	0.0379	0.0487	0.0354	0.0265
**7**	0.0302	0.0309	0.0309	0.0277	0.0259	0.0490	0.0328	0.0281
**8**	0.0279	0.0324	0.0296	0.0287	0.0314	0.0562	0.0306	−
**9**	0.0285	−	0.0319	−	0.0289	0.0579	−	−
**10**	0.0273	−	0.0312	−	0.0320	0.0564	−	−
**11**	0.0330	−	0.0316	−	0.0394	0.0582	−	−
**12**	0.0278	−	0.0302	−	−	−	−	−
**13**	0.0281	−	0.0295	−	−	−	−	−
**14**	−	−	0.0340	−	−	−	−	−
**15**	−	−	0.0295	−	−	−	−	−
**16**	−	−	0.0302	−	−	−	−	−
**17**	−	−	0.0310	−	−	−	−	−
**18**	−	−	0.0335	−	−	−	−	−
**19**	−	−	0.0301	−	−	−	−	−
**20**	−	−	0.0296	−	−	−	−	−

**Table A6:** Five best and worst randomsearch training results and respective hyperparameters.

Batch Size	Epochs	Neurons (HL)	Kernel Initializer (HL)	Activation (HL)	Dropout (HL)	Kernel Initializer (OL)	GD Optimizer	Mean MSE Parameter Train Set (−)
36	250	325	He Normal	Selu	0.05	Glorot Normal	Nadam	0.0317
21	125	300	He Normal	Softsign	0.25	Glorot Uniform	Adamax	0.0321
30	150	400	He Uniform	Tanh	0.15	He Uniform	Adamax	0.0322
18	75	450	He Normal	Selu	0.40	Glorot Normal	Adamax	0.0324
24	250	450	Uniform	Softsign	0.35	He Normal	Nadam	0.0329
21	100	250	Zero	Softmax	0.45	Lecun Uniform	Adadelta	0.0834
39	150	25	Lecun Uniform	Exponential	0.45	Normal	Adadelta	0.0844
15	150	75	Lecun Uniform	Softmax	0.05	Lecun Uniform	Adadelta	0.0920
33	250	450	Glorot Uniform	Softsign	0.05	Glorot Normal	Nadam	0.1009
15	150	350	Zero	Relu	0.10	Normal	Adadelta	0.1536

**Table A7:** Neural net settings from hyperparameteroptimization (* = default settings).

(Hyper-) Parameter	HPO1	HPO2	HPO3	HPO4	HPO5	HPO6	HPO7	HPO8
Batch Size	27	12	39	3	27	12	27	30
Epochs *	250	250	250	250	250	250	250	250
Neurons (IL) *	35	35	35	35	35	35	35	35
Number (Hidden) (Layer)	2	2	1	2	2	2	1	1
Neurons (HL1)	350	50	400	250	350	50	200	450
Kernel Initializer (HL1)	He Uniform	He Uniform	Lecun Uniform	He Uniform	He Uniform	He Uniform	He Uniform	Lecun Uniform
Activation (HL1)	Hard Sigmoid	Hard Sigmoid	Hard Sigmoid	Softsign	Hard Sigmoid	Hard Sigmoid	Hard Sigmoid	Selu
Dropout (HL1)	0.1	0.0	0.0	0.35	0.1	0.0	0.0	0.05
Neurons (HL2)	250	300	−	350	250	300	−	−
Kernel Initializer (HL2) *	normal	normal	normal	normal	normal	normal	normal	normal
Activation (HL2) *	linear	linear	linear	linear	linear	linear	linear	linear
Dropout (HL2) *	0.05	0.05	0.05	0.05	0.05	0.05	0.05	0.05
Neurons (OL) *	4	4	4	4	4	4	4	4
Kernel Initializer (OL)	He Uniform	Normal	He Uniform	He Normal	He Uniform	Normal	Glorot Uniform	Zero
Activation (OL) *	linear	linear	linear	linear	linear	linear	linear	linear
GD Optimizer	Adamax	Adam	Adam	Adagrad	Adamax	Adam	Adam	Adamax
HP Optimization Library	Keras Tuner	Keras Tuner	Keras Tuner	Hyperas	Keras Tuner	Keras Tuner	Keras Tuner	Hyperas
HP Optimizer	Random- search	Hyper- band	Bayesian	Bayesian TPE	Random- search	Hyper- band	Bayesian	Bayesian TPE
Max Trials *	100	100	100	100	100	100	100	100
Exekution per Trial *	2	2	2	2	2	2	2	2

## References

[1] Gibson I., Rosen D., Stucker B. (2015). Additive Manufacturing Technologies.

[2] Kumke M., Watschke H., Hartogh P., Bavendiek A.K., Vietor T. (2017). Methods and tools for identifying and leveraging additive manufacturing design potentials. Int. J. Interact. Des. Manuf. (IJIDeM).

[3] Mohamed O.A., Masood S.H., Bhowmik J.L. (2015). Optimization of fused deposition modeling process parameters: A review of current research and future prospects. Adv. Manuf..

[4] Mahnken R., Stein E. (1994). The identification of parameters for visco-plastic models via finite-element methods and gradient methods. Model. Simul. Mater. Sci. Eng..

[5] Mahnken R., Stein E. (1996). A unified approach for parameter identification of inelastic material models in the frame of the finite element method. Comput. Methods Appl. Mech. Eng..

[6] Morand L., Helm D. (2019). A mixture of experts approach to handle ambiguities in parameter identification problems in material modeling. Comput. Mater. Sci..

[7] Kučerová A., Zeman J. Estimating Parameters of Microplane Material Model Using Soft Computing Methods. Proceedings of the 6th World Congresses of Structural and Multidisciplinary Optimization.

[8] Goh G.D., Sing S.L., Yeong W.Y. (2020). A review on machine learning in 3D printing: Applications, potential, and challenges. Artif. Intell. Rev..

[9] Mehlig B. (2019). Artifical Neural Networks.

[10] Kučerová A. (2007). Identification of Nonlinear Mechanical Model Parameters Based on Softcomputing Methods. Ph.D. Thesis.

[11] Unger J.F., Könke C. (2011). An inverse parameter identification procedure assessing the quality of the estimates using Bayesian neural networks. Appl. Soft Comput..

[12] Soares C., de Freitas M., Araújo A., Pedersen P. (1993). Identification of material properties of composite plate specimens. Compos. Struct..

[13] Gelin J., Ghouati O. (1994). An inverse method for determining viscoplastic properties of aluminium alloys. J. Mater. Process. Technol..

[14] Araújo A., Soares C.M., de Freitas M. (1996). Characterization of material parameters of composite plate specimens using optimization and experimental vibrational data. Compos. Part B Eng..

[15] Fogel D. (1994). An introduction to simulated evolutionary optimization. IEEE Trans. Neural Netw..

[16] Yao L., Sethares W. (1994). Nonlinear parameter estimation via the genetic algorithm. IEEE Trans. Signal Process..

[17] Kerschen G., Worden K., Vakakis A.F., Golinval J.C. (2006). Past, present and future of nonlinear system identification in structural dynamics. Mech. Syst. Signal Process..

[18] Yagawa G., Okuda H. (1996). Neural networks in computational mechanics. Arch. Comput. Methods Eng..

[19] Jordan M.I., Rumelhart D.E. (1992). Forward Models: Supervised Learning with a Distal Teacher. Cogn. Sci..

[20] Huber N., Tsakmakis C. (1999). Determination of constitutive properties fromspherical indentation data using neural networks. Part i:the case of pure kinematic hardening in plasticity laws. J. Mech. Phys. Solids.

[21] Huber N., Tsakmakis C. (1999). Determination of constitutive properties fromspherical indentation data using neural networks. Part ii:plasticity with nonlinear isotropic and kinematichardening. J. Mech. Phys. Solids.

[22] Lefik M., Schrefler B. (2002). Artificial neural network for parameter identifications for an elasto-plastic model of superconducting cable under cyclic loading. Comput. Struct..

[23] Nardin A., Schrefler B., Lefik M. (2003). Application of Artificial Neural Network for Identification of Parameters of a Constitutive Law for Soils. Developments in Applied Artificial Intelligence, Proceedings of the 16th International Conference on Industrial and Engineering Applications of Artificial Intelligence and Expert Systems, IEA/AIE, Loughborough, UK, 23–26 June 2003.

[24] Helm D. (2005). Pseudoelastic behavior of shape memory alloys: Constitutive theory and identification of the material parameters using neural networks. Tech. Mech..

[25] Chamekh A., Salah H.B.H., Hambli R. (2008). Inverse technique identification of material parameters using finite element and neural network computation. Int. J. Adv. Manuf. Technol..

[26] Aguir H., Chamekh A., BelHadjSalah H., Dogui A., Hambli R. (2009). Parameter identification of a non-associative elastoplastic constitutive model using ANN and multi-objective optimization. Int. J. Mater. Form..

[27] MacKay D.J.C. (1992). Bayesian Interpolation. Neural Comput..

[28] Mareš T., Janouchová E., Kučerová A. (2016). Artificial neural networks in the calibration of nonlinear mechanical models. Adv. Eng. Softw..

[29] Livermore Software Technology Corporation (LSTC) LS-DYNA Keyword User’s Manual Volume II Material Models LS-DYNA, r11 ed.. https://www.dynamore.de/de/download/manuals/ls-dyna/ls-dyna-manual-r11.0-vol-ii-12-mb.

[30] Stander N.E.A. (2019). LS OPT User’s Manual—A Design Optimization and Probabilistic Analysis Tool for the Engeneering Analyst, v.6.0 ed.. https://www.lsoptsupport.com/documents/manuals/ls-opt/lsopt_60_manual.pdf.

[31] McKay M.D., Beckman R.J., Conover W.J. (1979). A Comparison of Three Methods for Selecting Values of Input Variables in the Analysis of Output from a Computer Code. Technometrics.

[32] Jin R., Chen W., Sudjianto A. (2005). An efficient algorithm for constructing optimal design of computer experiments. J. Stat. Plan. Inference.

[33] Fausett L., Fausett L. (1994). Fundamentals of Neural Networks: Architectures, Algorithms, and Applications.

[34] Gurney K. (2018). An Introduction to Neural Networks.

[35] Haykin S. (2009). Neural Networks and Learning Machines.

[36] Da Silva I.N., Spatti D.H., Flauzino R.A., Liboni L.H.B., dos Reis Alves S.F. (2017). Artificial Neural Networks.

[37] Shanmuganathan S., Samarasinghe S. (2016). Artificial Neural Network Modelling.

[38] Goodfellow I., Bengio Y., Courville A. (2016). Deep Learning.

[39] Pinto N., Doukhan D., DiCarlo J.J., Cox D.D. (2009). A High-Throughput Screening Approach to Discovering Good Forms of Biologically Inspired Visual Representation. PLoS Comput. Biol..

[40] Moons B., Bankman D., Verhelst M. (2019). Embedded Deep Learning.

[41] Hornik K., Stinchcombe M., White H. (1989). Multilayer feedforward networks are universal approximators. Neural Netw..

[42] Anders U., Korn O. (1999). Model selection in neural networks. Neural Netw..

[43] Nielsen M. (2015). Neural Networks and Deep Learning. http://static.latexstudio.net/article/2018/0912/neuralnetworksanddeeplearning.pdf.

[44] Hutter F., Kotthoff L., Vanschoren J. (2019). Automated Machine Learning.

[45] O’Malley T., Bursztein E., Long J., Chollet F., Jin H., Invernizzi L. Keras Tuner. https://github.com/keras-team/keras-tuner.

[46] Li L., Jamieson K.G., DeSalvo G., Rostamizadeh A., Talwalkar A. (2016). Efficient Hyperparameter Optimization and Infinitely Many Armed Bandits. arXiv.

[47] Bergstra J., Bardenet R., Bengio Y., Kegl B. (2011). Algorithms for Hyper-Parameter Optimization. Proceedings of the 24th International Conference on Neural Information Processing Systems ( NIPS’11).

[48] Falkner S., Klein A., Hutter F. (2018). BOHB: Robust and Efficient Hyperparameter Optimization at Scale. http://xxx.lanl.gov/abs/1807.01774.

[49] Bengio Y. (2012). Practical Recommendations for Gradient-Based Training of Deep Architectures. Neural Netw. Tricks Trade.

[50] Srivastava N., Hinton G., Krizhevsky A., Sutskever I., Salakhutdinov R. (2014). Dropout: A Simple Way to Prevent Neural Networks from Overfitting. J. Mach. Learn. Res..

